# Transcriptomic and Metabolomic Analyses Reveal That Fullerol Improves Drought Tolerance in *Brassica napus* L

**DOI:** 10.3390/ijms232315304

**Published:** 2022-12-04

**Authors:** Jun-Lan Xiong, Ni Ma

**Affiliations:** 1 Oil Crops Research Institute, Chinese Academy of Agricultural Science, Wuhan 430062, China; 2 School of Life Science, Lanzhou University, Lanzhou 730000, China

**Keywords:** fullerol, *Brassica napus*, drought, carbohydrate metabolism, biosynthesis of amino acids, phenolics and flavonoids

## Abstract

Carbon nanoparticles have potential threats to plant growth and stress tolerance. The polyhydroxy fullerene—fullerol (one of the carbon nanoparticles) could increase biomass accumulation in several plants subjected to drought; however, the underlying molecular and metabolic mechanisms governed by fullerol in improving drought tolerance in *Brassica napus* remain unclear. In the present study, exogenous fullerol was applied to the leaves of *B. napus* seedlings under drought conditions. The results of transcriptomic and metabolomic analyses revealed changes in the molecular and metabolic profiles of *B. napus*. The differentially expressed genes and the differentially accumulated metabolites, induced by drought or fullerol treatment, were mainly enriched in the Kyoto Encyclopedia of Genes and Genomes (KEGG) pathways related to carbohydrate metabolism (e.g., “carbon metabolism” and “galactose metabolism”), amino acid metabolism (e.g., “biosynthesis of amino acids” and “arginine and proline metabolism”), and secondary metabolite metabolism (e.g., “biosynthesis of secondary metabolites”). For carbohydrate metabolism, the accumulation of oligosaccharides (e.g., sucrose) was decreased, whereas that of monosaccharides (e.g., mannose and myo-inositol) was increased by drought. With regard to amino acid metabolism, under drought stress, the accumulation of amino acids such as phenylalanine and tryptophan decreased, whereas that of glutamate and proline increased. Further, for secondary metabolite metabolism, *B. napus* subjected to soil drying showed a reduction in phenolics and flavonoids, such as hyperoside and trans-3-coumaric acid. However, the accumulation of carbohydrates was almost unchanged in fullerol-treated *B. napus* subjected to drought. When exposed to water shortage, the accumulation of amino acids, such as proline, was decreased upon fullerol treatment. However, that of phenolics and flavonoids, such as luteolin and trans-3-coumaric acid, was enhanced. Our findings suggest that fullerol can alleviate the inhibitory effects of drought on phenolics and flavonoids to enhance drought tolerance in *B. napus*.

## 1. Introduction

Carbon-based nanomaterials such as fullerene, graphene, single-walled carbon nanotubes, and multi-walled carbon nanotubes are the most commonly used nanomaterials [1]. The unique physical, chemical, and mechanical properties of carbon nanotubes can provide solutions to various biological problems, particularly in the fields of biotechnology, medicine, pharmaceuticals, and agriculture [1,2]. The extensive production and application of carbon-based nanomaterials increases the chances of their release into biological cycles. Plants are a prominent part of the ecosystem and may act as a potential path for the uptake, translocation, and accumulation of nanoparticles into food chains; the environment is considered to comprise a large biomass that encounters released engineered nanomaterials [3]. Therefore, understanding plant responses to carbon nanomaterial exposure could open up new frontiers in agriculture, where continuous innovation is highly needed to guarantee global food security, and address environmental challenges.

Drought is considered as the most important environmental factor that limits crop growth and productivity worldwide [4]. It is important to raise environmental awareness and improve plant drought tolerance to sustainably enhance crop quality. A number of carbon nanomaterials are being investigated for use in agriculture to increase crop productivity, and protect crops from drought stress; one of the most investigated carbon nanomaterials is fullerene [5]. Some studies have reported positive effects associated with the application of fullerene under osmotic stress on plant growth in crop plants [5,6,7]. Fullerol (one of the water-soluble derivatives of fullerene) treatment at a concentration of 14 mg L^−1^ enhanced root growth in barley under 75 mM NaCl [6]. Fullerol treatment increased the leaf and root fresh weight in drought-treated sugar beets [8]. Exogenous fullerol administration by seed priming or foliar application stimulated growth in water-stressed *Brassica napus* [9]. 

*B. napus* is an important oilseed crop worldwide, and drought can impair its growth and grain yield [10]. Exploring the use of chemicals to increase drought tolerance is vital for the production of *B. napus*. Our previous work found that fullerol could promote drought tolerance in *B. napus* at the physiological level [9]. However, the effects of fullerol on drought resistance in *B. napus* at the molecular and metabolic levels are still unknown. RNA sequencing (RNA-seq) is a critical and suitable tool for gene expression analysis, using deep-sequencing technologies with high accuracy and sensitivity [11]. It is broadly applied to track transcriptomic variation in plants, in response to abiotic and biotic stresses. Moreover, metabolomic analysis provides valuable information on system-wide changes in plant metabolism, and allows for the identification of compounds with key roles in plant stress tolerance [12,13,14].

In this study, fullerol was applied to the leaves of seedlings subjected to drought stress in *B. napus*. We combined transcriptomic and metabolomic analyses to identify differences in gene transcript levels and metabolites, between non-fullerol-treated and fullerol-treated groups under water deficit conditions. We hypothesized that substantial differential gene expression and accumulation of differential metabolites existed in the fullerol-treated group, in comparison with the control group, under drought conditions. The aim of the present study was to determine whether fullerol affected drought tolerance at the molecular and metabolomic levels in *B. napus*.

## 2. Results

### 2.1. Aboveground Biomass and Leaf Relative Water Content

**Figure 1:**
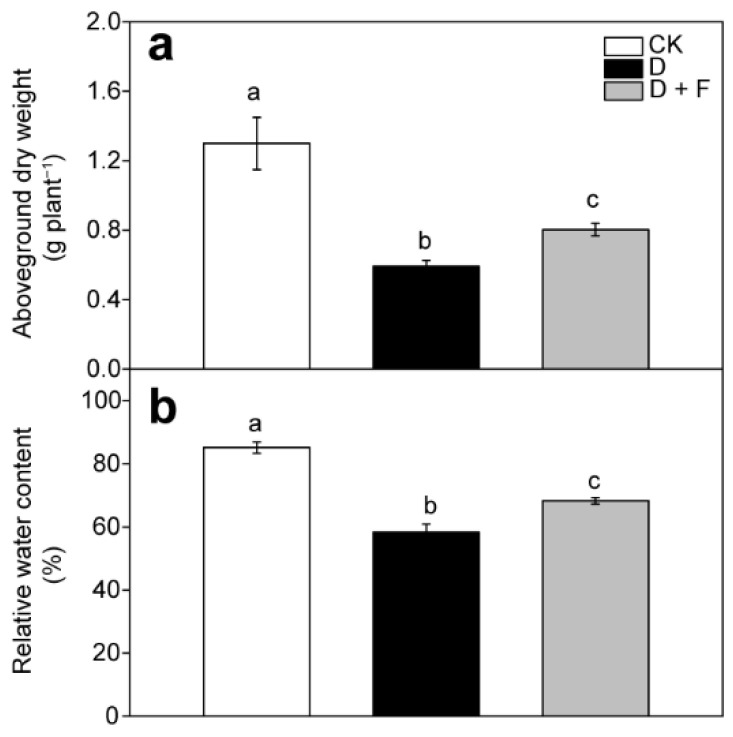
Aboveground biomass (**a**) and leaf relative water content (**b**) in leaves of *B. napus* treated with different fullerol treatments (0 and 100 mg L^−1^ F) and water gradients (CK: check, sufficient water condition; D: drought). Values are the means of three replicates ± standard error. The different letters in each subfigure indicate significant differences between water or fullerol treatments. Data were adapted from Xiong et al. [9].

Our previous work showed that water shortage significantly decreased the aboveground dry weight, as well as leaf relative water content (RWC) [9]. The drought-triggered decrease in aboveground biomass and leaf RWC were dramatically reversed by foliar application of fullerol with different concentrations (1, 10, and 100 mg L^−1^) [9]. Of these, the most effective concentration of fullerol was 100 mg L^−1^ [9]. Compared with leaves subjected to drought alone, those subjected to drought supplement with 100 mg L^−1^ fullerol treatment showed 35% and 25% increase in the aboveground dry weight and leaf RWC, respectively [9] (Figure 1). Because the most effective impact of fullerol on *B. napus* seedling subjected to soil drying was at the concentration of 100 mg L^−1^, we chose the leaves of *B. napus* treated with 100 mg L^−1^ fullerol to conduct transcriptomic and metabolic analyses.

### 2.2. Transcriptomic Analysis

#### 2.2.1. Analysis of Differentially Expressed Genes (DEGs)

Leaf tissues from *B. napus* under check (CK, sufficient water condition), drought (D), and drought with fullerol (D + F) treatments were obtained to construct three libraries for sequencing. From each of the three libraries, 54 to 64 million raw reads and 52 to 62 million clean reads were produced (Table S1). Approximately 84% of high-quality reads for each sample were mapped to a reference genome. Moreover, more than 46,000 transcripts with FPKM > 1 were identified in each library.

**Figure 2:**
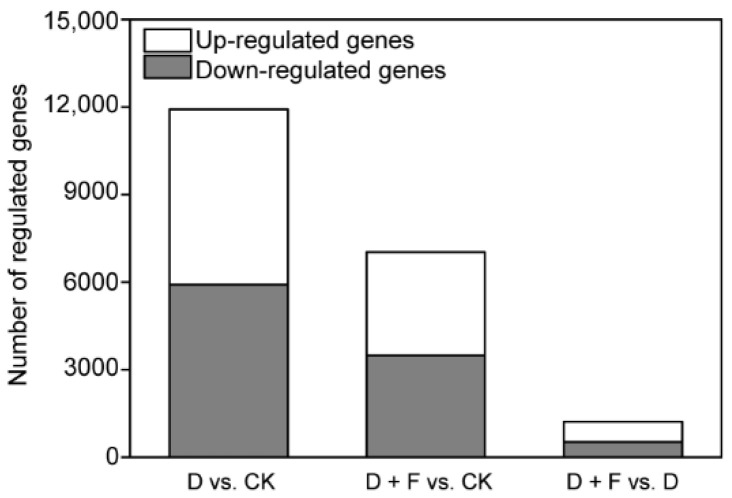
Summary of the numbers of differentially expressed genes (DEGs) in leaves of *B. napus* with drought and fullerol treatments. CK: check (sufficient water condition); D: drought; F: fullerol.

As shown in Figure 2, the comparison of different treatments identified 11,920, 7031, and 1222 DEGs in pairs of D vs. CK, D + F vs. CK, and D + F vs. D, respectively. Among them, 5917, 3494, and 529 genes were down-regulated and 6003, 3537, and 693 genes were up-regulated, respectively. In addition, 5968 DEGs were commonly regulated in D vs. CK and D + F vs. CK.

**Figure 3:**
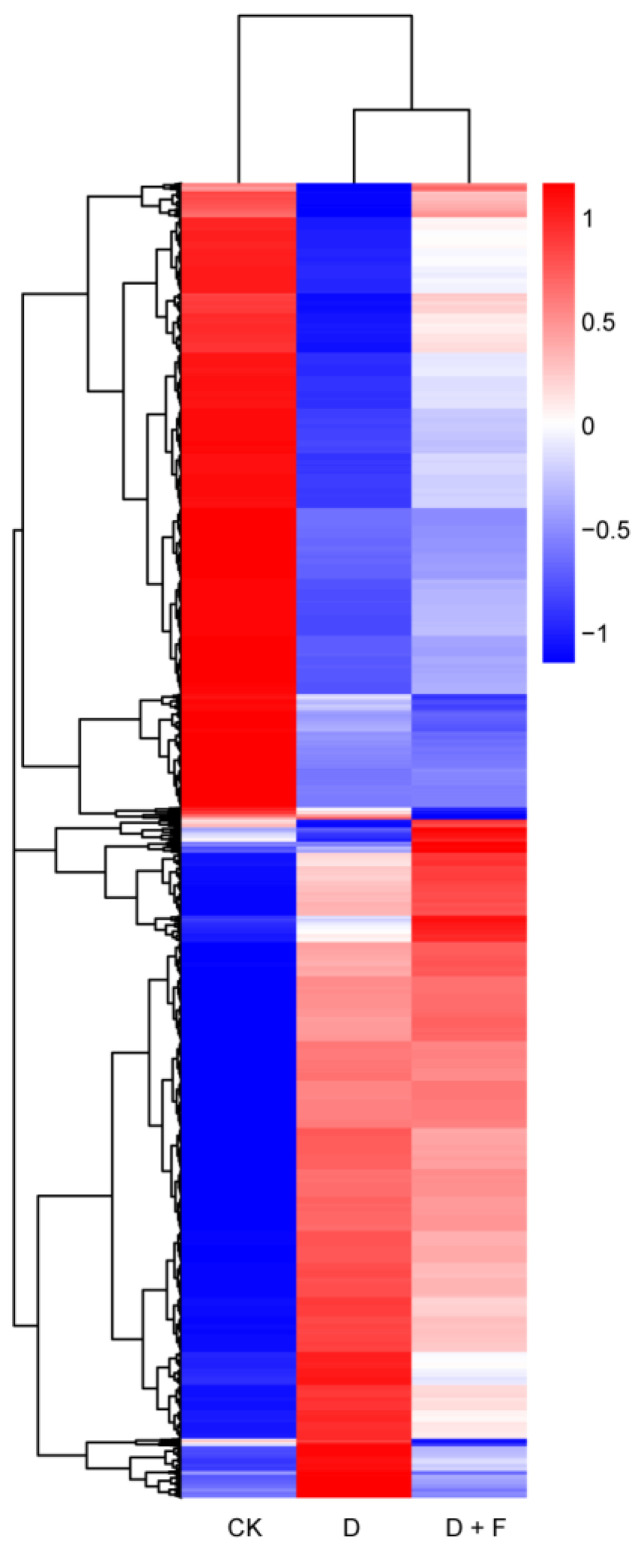
Hierarchical cluster analyses of differentially expressed genes in leaves of *B. napus* after drought and fullerol treatments. CK: check (sufficient water condition); D: drought; F: fullerol.

A heat map generated from the hierarchical clustering of DEGs is shown in Figure 3. The up-regulated and down-regulated genes between water and/or fullerol treatments are indicated by hierarchical clustering analysis. The expression pattern of DEGs in the D + F group was very similar to that of the D group, especially in the middle region of the heat map. In contrast, the expression pattern of DEGs in the D + F group was similar to that of the CK group at the end region of the heat map, which indicated that fullerol treatment reversed the inhibitory effect of drought on *B. napus* at the transcript level.

#### 2.2.2. Functional Analysis by Gene Ontology (GO) and Kyoto Encyclopedia of Genes and Genomes (KEGG)

**Figure 4:**
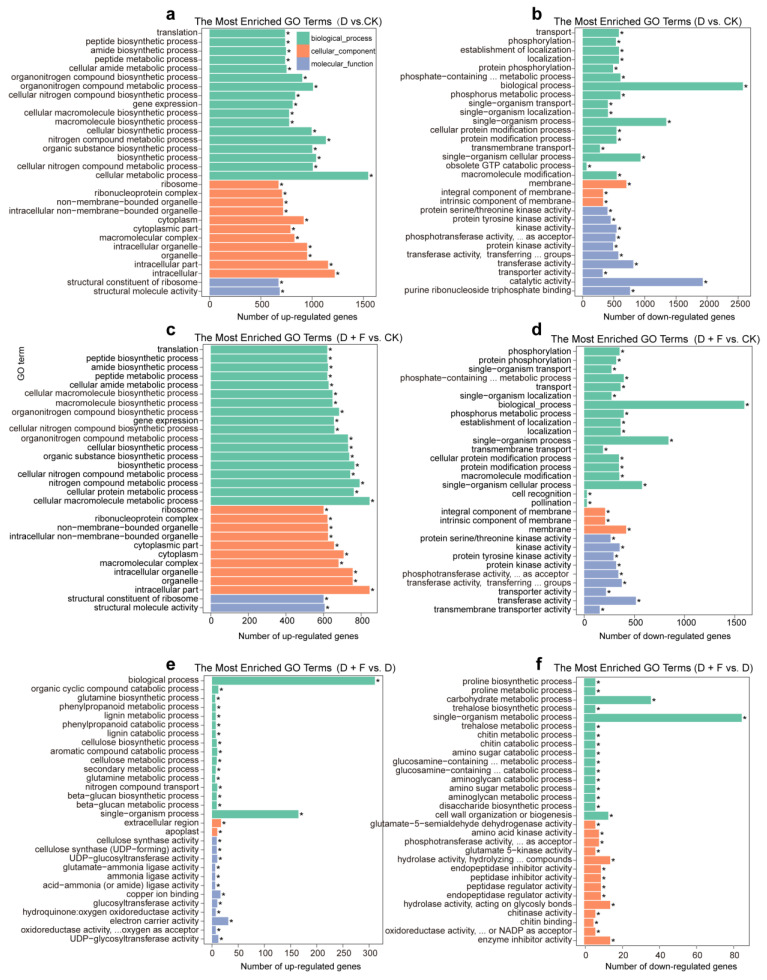
Gene ontology (GO) analysis of differentially expressed genes in leaves of *B. napus* after drought and fullerol treatments. The top 30 enriched GO categories for the up-regulated (**a**,**c**,**e**) and down-regulated (**b**,**d**,**f**) genes between drought (D) and check (CK, sufficient water condition) (D vs. CK), between drought with fullerol (D + F) and check (CK) (D + F vs. CK), and between non-fullerol-treated and fullerol-treated in the drought treatment (D + F vs. D), are given separately. “*” represents that the GO terms are significantly enriched at *p* < 0.05.

In drought vs. well-watered condition, 2687 GO terms were enriched in *B. napus* plants. Of these, the up-regulated genes induced by water deficit were significantly assigned to GO terms such as “peptide biosynthetic/metabolic process”, “organic substance biosynthetic process”, and “nitrogen compound metabolic process” (Figure 4a). The down-regulated genes induced by drought treatments were markedly assigned to GO terms such as “protein serine/threonine kinase activity”, “protein kinase activity”, and “transport” (Figure 4b).

In the drought addition with the fullerol group vs. the well-watered group, DEGs were enriched in 2287 GO terms. The up-regulated genes triggered by drought with fullerol treatment were dramatically assigned to GO terms such as “ribosome”, “peptide biosynthetic process”, “structural molecule activity”, and “macromolecule biosynthetic process” (Figure 4c). The down-regulated genes caused by the drought with fullerol treatment were significantly enriched in GO terms such as “phosphorylation”, “protein serine/threonine kinase activity”, “protein phosphorylation”, and “phosphate-containing compound metabolic process” (Figure 4d).

The DEGs between the D + F group and the D group were analyzed and assigned to 1099 GO terms. Among them, the up-regulated genes caused by fullerol treatment under drought stress were mainly assigned to GO terms such as “organic cyclic compound catabolic process”, “cellulose synthase activity”, “glutamine biosynthetic process”, and “phenylpropanoid metabolic process” (Figure 4e). The down-regulated genes were assigned to GO terms such as “amino acid kinase activity”, “glutamate-5-semialdehyde dehydrogenase activity”, “proline metabolic process”, “carbohydrate metabolic process”, and “single-organism metabolic process” (Figure 4f).

Compared with well-watered conditions, 119 KEGG pathways were enriched under drought conditions. The up-regulated genes were significantly enriched in KEGG pathways such as “biosynthesis of amino acids”, “2-Oxocarboxylic acid metabolism”, “carbon fixation in photosynthetic organs”, and “arginine and proline metabolism” (Figure S1a). The down-regulated genes were enriched in KEGG pathways such as “amino sugar and nucleotide sugar metabolism”, “starch and sucrose metabolism”, and “plant hormone signal transduction” (Figure S1b). 

In the drought with fullerol treatment vs. sufficient water condition, 116 KEGG pathways were enriched in the leaves of *B. napus* seedlings. The up-regulated genes were significantly assigned to KEGG pathways such as “biosynthesis of amino acids”, “galactose metabolism”, “arginine and proline metabolism”, and “tryptophan metabolism” (Figure S1c). The down-regulated genes were significantly enriched in KEGG pathways such as “phosphatidylinositol signaling system”, “fatty acid biosynthesis”, “arginine and proline metabolism”, and “starch and sucrose metabolism” (Figure S1d). 

The DEGs induced by fullerol under water stress were assigned to 93 KEGG pathways, compared to drought alone. Among them, the up-regulated genes were assigned to KEGG pathways such as “starch and sucrose metabolism”, “biosynthesis of amino acids”, “biosynthesis of secondary metabolism”, “flavonoid biosynthesis”, and “phenylalanine metabolism” (Figure S1e). The down-regulated genes were enriched in KEGG pathways such as “starch and sucrose biosynthesis”, “biosynthesis of amino acids”, “arginine and proline metabolism”, and “biosynthesis of secondary metabolism” (Figure S1f).

#### 2.2.3. Quantitative Real-Time (qRT)-PCR

**Figure 5:**
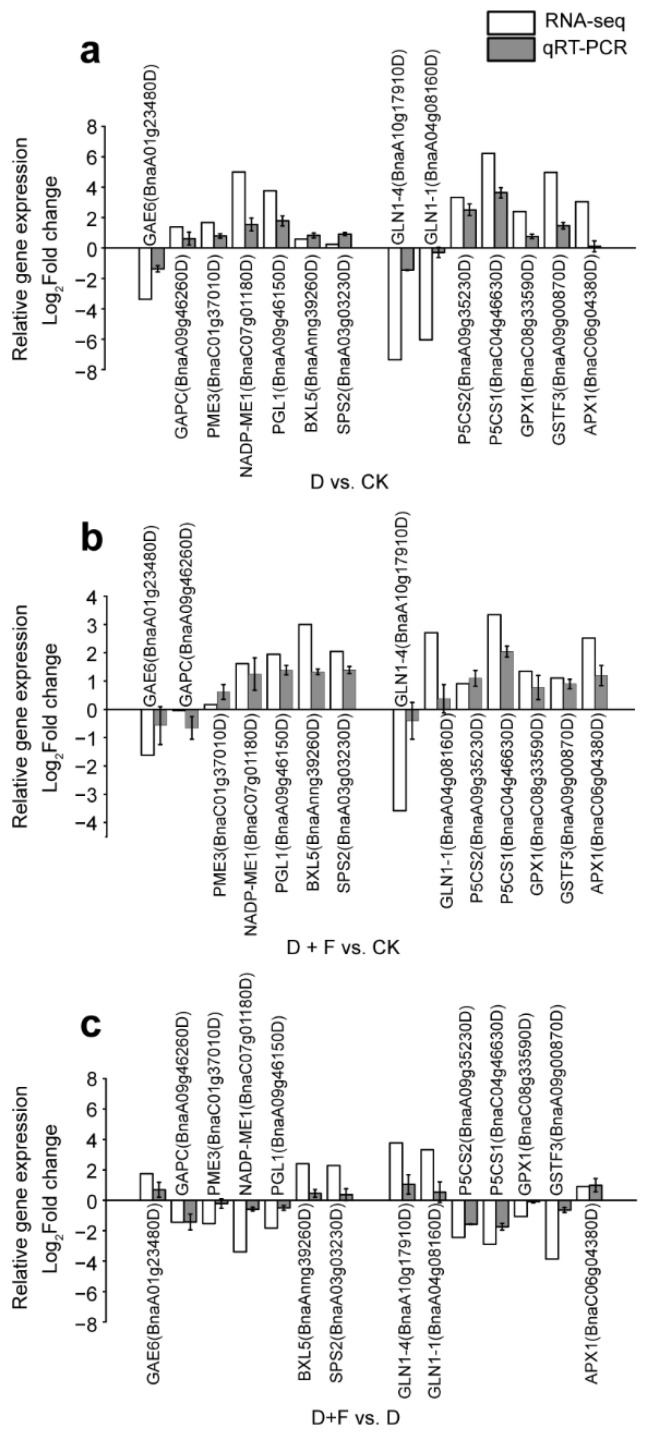
Validation of the expression levels of differentially expressed genes obtained from RNA-seq analyses using qRT-PCR. CK: check (sufficient water condition); D: drought; F: fullerol. (**a**) Group D vs. CK. (**b**) Group D + F vs. CK. (**c**) Group D + F vs. D. Values are the means of three replicates ± standard error.

We conducted qRT-PCR to validate the RNA-seq data and analyze gene expression changes of randomly selected genes. These selected genes that were orthologous to genes in *Arabidopsis thaliana* were mainly associated with carbohydrate metabolism (*GAPC*, *PME3*, *NADP-ME2*, *PGL1*, *BXL5*, *SPS2*, and *GAE6*) and drought response (*GPX1*, *GSTF3*, *APX1*, *GLN1-1*, *GLN1-4*, *P5CS1*, and *P5CS2*) (Figure 5). Although the expression levels of selected genes in D vs. CK, D + F vs. CK, or D + F vs. D were different between RNA-seq and qRT-PCR, the expression patterns of DEGs obtained from RNA-seq were similar to those of genes obtained from qRT-PCR (Figure 5).

### 2.3. Metabolic Analysis

A separation trend was observed among the sufficient water condition (CK), drought (D), and drought combined with fullerol (D + F) treatments using principal component analysis (PCA), indicating that drought and fullerol had an impact on the *B. napus* metabolism. The CK group and D group (Figure S2a), the CK group and D + F group (Figure S2b), and the D group and D + F group (Figure S2c) were distinguished. In addition, 77, 74, and 62 metabolites were significantly identified to be responsible for the separation in D vs. CK, D + F vs. CK, and D + F vs. D, respectively (Supplementary Materials Files S1–S3). Among them, 41 metabolites exhibited an increased pool size, and 36 metabolites showed a decreased pool size in D vs. CK (Supplementary Materials File S1); 47 metabolites were increased, and 27 metabolites were decreased in D + F vs. CK (Supplementary Materials File S2); and 20 metabolites were increased, and 42 metabolites were decreased in D + F vs. D (Supplementary Materials File S3). The detected compounds comprised sugars or their derivates, amino acids, nitrogen-containing compounds, phenolics and flavonoids, and others (Supplementary Materials Files S1–S3).

Linking tentatively identified metabolites to biochemical pathways can aid in targeting key changes, as the constituents of a given pathway are likely to be co-regulated. Metabolites were mainly enriched in KEGG pathways such as “biosynthesis of secondary metabolites”, “biosynthesis of antibiotics”, “ABC transporters”, “biosynthesis of amino acids”, and “carbon metabolism” in all three groups (D + F vs. D, D + F vs. CK, and D vs. CK) (Supplementary Materials Files S4–S6).

### 2.4. Data Integration/Comprehensive Networks of Transcripts and Metabolites

With the aim of characterizing the progression of fullerol in *B. napus* in response to drought, we performed transcriptomic and metabolic data integration based on common KEGG pathways. Data integration detected the most important biological processes on the basis of KEGG pathway under water stress or fullerol treatments in *B. napus*.

Using combined transcriptomic and metabolomic data, we found that DEGs and differentially accumulated metabolites were commonly enriched in 48, 43, and 36 KEGG pathways in D vs. CK, D + F vs. CK, and D + F vs. D, respectively (Supplementary Materials Files S7–S9). Among them, KEGG pathways such as “biosynthesis of secondary metabolites”, “biosynthesis of amino acids”, “carbon metabolism”, and “galactose metabolism” were identified to have the most genes and metabolites (Supplementary Materials Files S7–S9) in D vs. CK, D + F vs. CK, and D + F vs. D groups. We also aimed to investigate flavonoid metabolism associated with antioxidant ability, in response to drought. Therefore, modifications at the transcriptomic and metabolomic levels were analyzed in detail for the following biochemical processes: carbohydrate metabolism, amino acid metabolism, and secondary metabolite metabolism. Partial genes related to these biochemical processes such as *GLN1-1*, *GLN1-4*, *P5CS1*, and *P5CS2* were validated by qRT-PCR (Figure 5).

#### 2.4.1. Carbohydrate Metabolism

**Figure 6:**
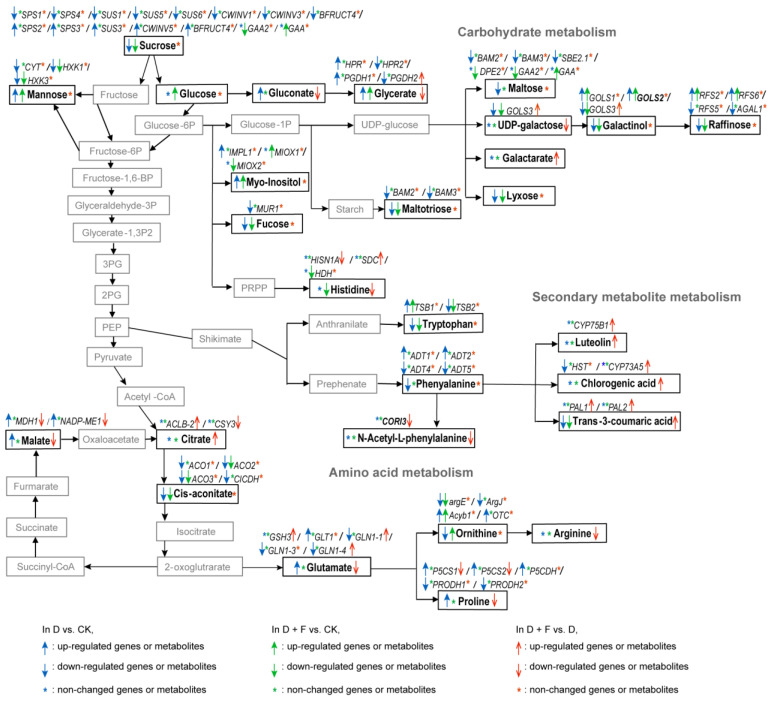
Map of genes and metabolites enriched in common KEGG pathways related to carbohydrate metabolism, amino acid metabolism, and biosynthesis of plant secondary metabolites in a comparison between drought (D) and check (CK, sufficient water condition), between drought with fullerol treatment (D + F) and check (CK), or between drought with fullerol treatment (D + F) and drought alone (D). Downward-pointing arrows indicate that the genes or metabolites are down-regulated, and upward-pointing arrows indicate that the genes or metabolites are up-regulated. The blue arrows indicate changes in genes or metabolites occurring in the D vs. CK group, the green arrows indicate changes in genes or metabolites occurring in the D + F vs. CK group, and the red arrows indicate changes occurring in the D + F vs. D group. The differentially expressed genes with assay names of the gene ID are given in Supplementary Materials File S10.

Under drought stress, the accumulation of oligosaccharides was inhibited, whereas that of monosaccharides was promoted. In the “starch and sucrose metabolism” pathway, the contents of oligosaccharides, including sucrose and maltose, were decreased in drought-treated plants compared to well-water-treated plants (Figure 6). Genes encoding the sucrose biosynthetic enzymes, including probable sucrose-phosphate synthase (e.g., *SPS1*/*SPS4*) and sucrose synthase (e.g., *SUS1*/*SUS5*/*SUS6*), were partially down-regulated, while genes encoding the sucrose catabolic enzymes, including β-fructofuranosidase (e.g., *CWINV5*) and acid β-fructofuranosidase (e.g., *BFRUCT4*), were partially up-regulated by water deficit (Figure 6). The transcript abundances of enzymes involved in maltose biosynthesis, such as β-amylase (*BAM2*/*BAM3*) and 1,4-α-glucan-branching enzyme 2-1 (*SBE2.1*), were decreased by drought stress (Figure 6). In the “galactose metabolism” pathway, the galactinol and raffinose (oligosaccharide) contents were decreased by drought (Figure 6). Among them, galactinol is the mediate product of oligosaccharides. Galactinol synthase (GOLS) can catalyze the conversion of UDP-galactose into galactinol, and the gene encoding galactinol synthase (e.g., *GOLS3*) was partially down-regulated in drought-treated plants (Figure 6). The expression of genes encoding raffinose biosynthetic enzymes, including probable galactinol-sucrose galactosyltransferase (e.g., *RFS5*) and α-galactosidease 1 (*AGAL1*), was partially down-regulated by drought (Figure 6). Here, both the “starch and sucrose metabolism” and “galactose metabolism” pathways belong to carbohydrate metabolism. Other oligosaccharides including maltotriose, lyxose, and fucose were also decreased in plants undergoing water stress. The genes *BAM2* and *BAM3* related to maltotriose biosynthesis, as well as the gene encoding GDP-mannose 4, 6 dehydratase (*MUR1*) associated with fucose biosynthesis, were down-regulated in drought-exposed plants (Figure 6). In contrast, water deficit improved the accumulation of monosaccharides and their derivates in *B. napus*. Drought caused increases in the contents of mannose and glycerate (monosaccharide). The expression of genes encoding hexokinase (*HXK1* and *HXK3*), related to mannose catabolism, were down-regulated by drought (Figure 6). The expression of genes encoding glycerate dehydrogenase (e.g., *HPR2*) and D-3-phosphoglycerate dehydrogenase (e.g., *PGDH2*) associated with glycerate catabolism were partially down-regulated in *B. napus* under drought (Figure 6). Water deficit led to an increment in the content of gluconate, which is a derivate of glucose (monosaccharide). Myo-inositol, a monosaccharide-like substance, is a kind of soluble sugar alcohol whose content was elevated by drought. The phosphatase IMPL1 can catalyze the conversion of myo-inositol phosphate into myo-inositol, and the expression of the gene *IMPL1* was up-regulated under drought (Figure 6).

Drought with fullerol treatment decreased the accumulation of oligosaccharides (e.g., sucrose, maltotriose, raffinose, fucose, lyxose, and galactinol) and increased the accumulation of monosaccharides (e.g., glucose, glycerate, mannose, and myo-instiol), compared to sufficient water conditions (Figure 6). At the transcript level, for oligosaccharides, drought addition with fullerol up-regulated the expression of gene encoding alpha-glucosidase (*GAA*) associated with sucrose catabolism, and down-regulated the expression level of gene (*GOLS3*) related to galactinol synthase—in comparison with well-watered condition (Figure 6). For monosaccharides, the expression of genes (*HXK1* and *HXK3*) related to mannose catabolism, and the expression of the gene encoding myo-Inositol oxygenase 2 (*MIOX2*), which catalyzes myo-inositol catabolism, were down-regulated in the D + F vs. CK group (Figure 6). 

In drought supplementation with the fullerol group vs. drought alone (D + F vs. D), the accumulation of monosaccharides was changed, while the accumulation of oligosaccharides showed almost no change (Figure 6). The contents of the derivate of galactose, UDP-galactose, and the derivate of glucose, gluconate, were reduced in the fullerol treatment in *B. napus* under water deficit, where the galactose and glucose belong to monosaccharides (Figure 6). Galactinol synthase 3 (GOLS3) can catalyze the conversion of UDP-galactose into galactinol, and drought with fullerol triggered a high expression of gene *GOLS3*, compared to drought alone (Figure 6). In contrast, another galactose derivative, galactarate, was increased by fullerol in drought-treated *B. napus*. However, the glycerate content was lower, and the transcript level of the gene *PGDH2* associated with glycerate catabolism was higher, in fullerol-treated plants than in non-fullerol-treated plants under drought treatment (Figure 6).

In the tricarboxylic acid (TCA) cycle (involved in carbohydrate metabolism), water stress decreased the content of cis-aconitate and increased the content of malate, compared to well-watered conditions (Figure 6). The expression of biosynthetic genes *ACO1*/*ACO2*/*ACO3* (encoding aconitase) and the expression of catabolic gene *CICDH* (encoding isocitrate dehydrogenase) for cis-aconitate were inhibited by drought (Figure 6). The expression of the gene encoding malate dehydrogenase 1 (*MDH1*) which catalyzes the conversion of oxaloacetate to malate, and the expression of gene encoding NADP-malic enzyme 1 (*NADP-ME1*) which can degrade malate, were up-regulated by water deficit (Figure 6). In addition, in comparison with sufficient water conditions, drought addition with fullerol treatment caused a reduction in the content of cis-aconitate and down-regulated its biosynthetic genes *ACO2*/*ACO3*. There was no significant difference in the malate content between well-watered treatment and drought with fullerol treatment (Figure 6).

Compared with the drought alone, in the TCA cycle, the citrate content was increased, and the malate content was decreased in the drought supplementation with fullerol treatment (Figure 6). Fullerol application increased the expression of the gene (*ACLB-2*) encoding ATP-citrate synthase beta chain protein 2, related to citrate biosynthesis, under drought stress (Figure 6). The expression of the genes *MDH1* and *NADP-ME1* associated with malate synthesis was repressed by fullerol treatment under drought (Figure 6).

#### 2.4.2. Amino Acid Metabolism

Under drought stress, several amino acids derived from the shikimate pathway (mainly in “biosynthesis of phenylpropanoids” pathway), including phenylalanine and tryptophan, were decreased in the leaves of drought-treated *B. napus* seedlings in comparison with well-water-treated plants (Figure 6). The arogenatedehydratase genes (e.g., *ADT4*/*ADT5*) associated with phenylalanine biosynthesis were partially down-regulated under drought (Figure 6). For tryptophan biosynthesis, plants exposed to a water deficit partially down-regulated the expression of the tryptophan synthase gene (e.g., *TSB2*) (Figure 6).

The biosynthesis of amino acids from the 2-oxoglutarate pathway (mainly in the “arginine and proline metabolism” pathway) had different patterns in response to drought: the levels of glutamate and proline were increased, while the ornithine content was decreased (Figure 6). For glutamate, the gene encoding glutamate synthase 1 (*GLT1*), associated with glutamate biosynthesis, was up-regulated, while the genes encoding glutamine synthetase cytosolic isozyme (*GLN1-1*/*GLN1-3*/*GLN1-4*), related to glutamate catabolism, were down-regulated by drought—when compared to sufficient water conditions (Figure 6). For proline, two delta-1-pyrroline-5-carboxylate synthase genes (*P5CS1*/*P5CS2*), related to proline synthesis, were increased, while genes (*PRODH1*/*PRODH2*) encoding proline dehydrogenase, associated with proline catabolism, were suppressed by water stress (Figure 6). In terms of ornithine biosynthetic genes, one acetylornithine deacetylase gene (*argE*) and one arginine biosynthesis bifunctional protein gene (*ArgJ*) were suppressed, while one aminoacylase-1B gene (*Acyb1*) was increased by drought (Figure 6). Water shortage up-regulated the expression of the ornithine catabolism gene encoding ornithine carbamoyltransferase (*OTC*) (Figure 6).

In drought with fullerol treatment vs. sufficient water condition (D + F vs. CK), the accumulation of amino acids in the leaves of *B. napus* seedlings was changed. For amino acids derived from the shikimate pathway, drought addition with fullerol treatment did not change the content of phenylalanine, but showed a reduction in tryptophan content as well as partially repressed the expression of the tryptophan synthetic gene (e.g., *TSB2*), when compared to well-watered conditions (Figure 6). For amino acids derived from the 2-oxoglutarate pathway, drought with fullerol treatment had almost no effects on the contents of glutamate and proline, but increased the ornithine content and up-regulated the expression of gene *Acyb1,* related to ornithine biosynthesis, in comparison with sufficient water conditions (Figure 6). For amino acids derived from phosphoribosyl pyrophosphate, in the D + F vs. CK group, the content of histidine was decreased and the expression of the gene encoding histidinol dehydrogenase (*HDH*), which oxidizes histidinol to histidine, was down-regulated (Figure 6).

Compared with drought alone, drought with fullerol treatment had no impacts on amino acids from the shikimate pathway, except for N-acetyl-phenylalanine (Figure 6). The N-acetyl-phenylalanine level and its biosynthetic gene *CORI3* were suppressed by fullerol under water stress (Figure 6). The contents of amino acids (glutamate, proline, and arginine) derived from the 2-oxoglutarate pathway were decreased in fullerol-treated plants, in comparison with non-fullerol-treated plants, under drought. At the transcript levels, for glutamate, one catabolic gene encoding probable glutamate dehydrogenase 3 (*GSH3*) and two catabolic genes (*GLN1-4*/*GLN1-1*) were up-regulated in fullerol-treated plants under drought. With regard to proline, two biosynthetic genes *P5CS1*/*P5CS2* were suppressed by fullerol in *B. napus* subjected to soil drying (Figure 6). However, exogenous fullerol decreased the amino acids from phosphoribosyl pyrophosphate, including histidine and histamine, under drought (Figure 6). The transcript level of the gene encoding ATP phosphoribosyltransferase 1 (*HISN1A*), related to histidine synthesis, was lower and the transcript level of gene encoding serine decarboxylase (*SDC*), associated with histidine catabolism, was higher, in fullerol-treated plants than non-fullerol-treated plants under water shortage conditions (Figure 6).

#### 2.4.3. Secondary Metabolite Metabolism

Under drought stress, plants can produce a variety of secondary metabolites; of these, phenolics and flavonoids are substances with antioxidant capacity. *B. napus* plants undergoing drought stress had a reduction in most detected phenolics and flavonoids, including hyperoside, peonidin 3-O-glucoside cation, quercetin 3′-methyl ether, 3-hydroxy-4-methoxycinnamic acid, and trans-3-coumaric acid, compared to sufficient water conditions (Supplementary Materials File S1 and Figure 6). Of these, trans-3-coumaric acid can be mapped to “phenylalanine metabolism” of the KEGG pathway (Supplementary Materials File S7 and Figure 6).

Compared with the well-watered conditions, drought with fullerol treatment also decreased the contents of phenolics and flavonoids, including peonidin 3-O-glucoside cation, quercetin 3′-methyl ether, 3-hydroxy-4-methoxycinnamic acid, and trans-3-coumaric acid (Supplementary Materials File S2 and Figure 6). The values of log_2_fold change in most of the detected phenolics and flavonoids were higher in the D + F vs. CK group than in the D vs. CK group (Supplementary Materials Files S1 and S2).

Drought addition with fullerol led to an increase in the accumulation of most detected phenolics and flavonoids, including luteolin, rutin, chlorogenic acid, trans-3-coumaric acid, and 3-hydroxy-4-methoxycinnamic acid, compared to drought stress alone (Supplementary Materials File S2 and Figure 6). Among them, chlorogenic acid and luteolin can be mapped to “flavonoid biosynthesis” of the KEGG pathway (Supplementary Materials File S9 and Figure 6). The gene encoding flavonoid 3′-monooxygenase (*CYP75B1*), associated with luteolin biosynthesis, was up-regulated by fullerol in *B. napus* subjected to water deficit (Figure 6). Foliar application of fullerol elevated the expression of genes encoding phenylalanine ammonia-lyase (*PAL1*/*PAL2*) for trans-3-coumaric acid biosynthesis in drought-treated *B. napus* (Figure 6).

## 3. Discussion

*B. napus* is a critical oil crop grown worldwide, and water deficit poses a threat to its growth and yields. Fullerol is a small-sized carbon nanoparticle with high amounts of polyhydroxy fullerenes, exhibiting positive effects on *B. napus* under drought stress at the physiological level. However, the mechanisms of fullerol at the molecular and metabolic levels in *B. napus* in response to drought remain unclear. In this study, we used transcriptomic and metabolomic analyses to identify differentially expressed genes and differentially accumulated metabolites caused by drought or fullerol; consequently, the molecular and metabolic mechanisms of *B. napus* subjected to fullerol under drought were investigated.

When exposed to soil drying, plants can maintain basal metabolic activities through a series of molecular and biochemical adaptations. In this study, RNA-seq analysis showed that the expression profiles of a large number of DEGs in *B. napus* were altered by drought. Functional enrichment of these DEGs presented that drought triggered KEGG pathways such as “biosynthesis of amino acids”, “carbon fixation in photosynthetic organs”, “arginine and proline metabolism”, and “starch and sucrose metabolism”. In addition, plants can dramatically accumulate metabolites under drought stress. Previous studies have shown that plants can accumulate several metabolites such as sugars, amino acids, organic acids, nucleotides and their derivatives, and phenolics and flavonoids to regulate intracellular osmotic pressure, and scavenge reactive oxygen species (ROS) in response to drought [15,16]. In the present study, metabolomic analysis revealed that drought stress induced a variety of metabolites such as sugars, amino acids, organic acids, and their derivatives, in *B. napus* subjected to drought. The KEGG pathway enrichment analysis revealed that the detected metabolites were mainly enriched in metabolic pathways related to “biosynthesis of amino acids”, “biosynthesis of secondary metabolites”, and “carbon metabolism”. These results were consistent with those of previous studies conducted by Zhao et al. [15], Xiong et al. [17], and Vital et al. [18].

The foliar application of fullerol could induce genes and metabolites that were differentially expressed and differentially accumulated in *B. napus* under drought stress. The most enriched KEGG pathways from DEGs and metabolites induced by fullerol in *B. napus* under drought were similar to those of the drought control. These pathways were mainly concentrated in “starch and sucrose metabolism”, “carbon metabolism”, “galactose metabolism”, “biosynthesis of amino acids”, “arginine and proline metabolism”, etc. Among them, “starch and sucrose metabolism”, “carbon metabolism”, and “galactose metabolism” were related to carbohydrate metabolism. KEGG pathways such as “biosynthesis of amino acids” and “arginine and proline metabolism” were associated with amino acid metabolism. Additionally, the antioxidant-related KEGG pathways such as “flavonoid biosynthesis” and “phenylalanine metabolism” were enriched. Therefore, by comparing the transcriptome and metabolome results, we concluded that fullerol mainly affected the KEGG pathways related to carbohydrate metabolism, amino acid metabolism, and secondary metabolite metabolism, at the molecular and metabolic levels in *B. napus* under drought. Here, we would explore the mechanisms of the effects of fullerol on drought adaptation in *B. napus* based on these three biochemical processes.

### 3.1. Carbohydrate Metabolism

Our previous studies indicated that dry matter (carbohydrate) accumulation in *B. napus* was reduced by drought [9]. The present study further revealed that water deficit decreased the contents of oligosaccharides (related to dry matter accumulation), and down-regulated the expression of genes associated with oligosaccharide biosynthesis (or up-regulated the expression of genes involved in oligosaccharide catabolism). Metabolomic analysis showed that the contents of oligosaccharides including sucrose, fucose, raffinose, and maltose were decreased by drought. Transcriptome analysis supported the metabolomic results and indicated that water shortage depressed the expression of several genes associated with the biosynthesis of oligosaccharides, such as sucrose synthase gene *SPS1*/*SPS4* and raffinose synthesis gene *RFS5*/*AGAL1*. Several studies were consistent with our results. For example, Rahman et al. [19] showed that the contents of sucrose and raffinose in wheat were decreased under post-anthesis drought stress. Drought reduced the sucrose concentration in soybean [20]. In contrast, water shortage improved the accumulation of monosaccharides, and up-regulated the expression of genes related to monosaccharide biosynthesis (or down-regulated genes involved in monosaccharide catabolism). The contents of the monosaccharide, including mannose and glycerate, as well as the content of monosaccharide analogue (myo-inositol), were increased in leaves of *B. napus* under drought stress. Related genes such as the myo-inositol synthesis gene, *IMPL1*, were also increased by water deficit. Previous studies agreed with these results, and Mutwakil et al. [21] reported that a sharp increase in myo-inositol was found in *Calotropis procera* subjected to salt and drought stress. The glycerate level was elevated in both *Ulmus minor* Mill. and *Quercus ilex* L. seedlings under drought [22]. These findings were in accordance with Rodríguez-Calcerrada et al. [23], who stated that simple sugars and sugar alcohols presented a significant increase, whereas compound sugars (e.g., sucrose) decreased or did not change under severe drought stress conditions. The oligosaccharides are energetic and structural substances that can serve as carbon sources for plant growth and development [24,25], while the monosaccharides can act as stress regulators for drought adaptation in plants [26,27]. For example, mannose can be involved in osmoregulation as a low molecular sugar in plants [26]. Myo-inositol can serve as an important stress regulator, both as a key metabolite to regulate osmotic balance and scavenge ROS [27]. Under drought stress, oligosaccharides can be broken down into monosaccharides with lower molecular weights to increase the osmotic potential of cells [24,25]. Therefore, we can speculate that *B. napus* exposed to drought may decompose oligosaccharides into low-molecular sugars such as monosaccharides, which can enhance osmotic adjustment capacity, and even scavenge ROS for adaptation to drought.

Under drought stress, exogenous application of fullerol resulted in almost no changes in the oligosaccharide contents. For monosaccharides, fullerol exhibited inconsistent changes in a few monosaccharides, including glycerate and the derivatives of galactose, in drought-treated plants. As an example, the changes in the derivatives of galactose caused by fullerol under drought were different: decreasing the content of UDP-galactose, and increasing the content of galactarate. These results implied that fullerol may not induce the accumulation of monosaccharides to enhance osmotic adjustment capacity in *B. napus* under drought. 

### 3.2. Amino Acid Metabolism

Amino acid metabolism is the main component of nitrogen metabolism, and in this study, drought mainly affected the accumulation of amino acids derived from the 2-oxoglutarate and shikimate pathways. For the 2-oxoglutarate pathway, our study found that water deficit induced glutamate and proline accumulation, but decreased ornithine formation, which agreed with the findings of Hatzig et al. [28]. The expression of genes encoding proline synthesis (*P5CS1*/*P5CS2*) was up-regulated, and the expression of genes encoding proline catabolism (*PRODH1*/*PRODH2*) was down-regulated by drought. Water stress also up-regulated the expression of the ornithine catabolism gene (*OTC*). Among them, proline is an important osmotic adjustment substance and ROS scavenger, and its accumulation can help to maintain water in plants under osmotic stress, and regulate the redox status of cells [29,30]. In this study, the increase in proline may help to maintain water potential and scavenge ROS in *B. napus*, in response to drought. In contrast, ornithine was found to be decreased under drought. This may be due to the fact that glutamate is a common precursor for proline and ornithine synthesis. In comparison with ornithine, proline synthesis from glutamate is predominant under stress conditions, and the high requirements for proline synthesis may limit ornithine synthesis [28]. 

For the shikimate pathway, drought decreased the contents of phenylalanine and tryptophan. The genes *ADT4*/*ADT5*, which are associated with the biosynthesis of phenylalanine, and the gene *TSB2*, which is related to tryptophan biosynthesis, were down-regulated in *B. napus* subjected to drought. Although most studies pointed out that the accumulation of phenylalanine and tryptophan can be enhanced by water deficit [31,32], several studies showed opposite results, and agreed with our findings. Khan et al. [33] reported that a decrease in phenylalanine level was found in chickpea exposed to drought. Here, phenylalanine is a biosynthetic precursor of phenolics and flavonoids, which can play an antioxidant role in the plant defense system [34,35]. The reduction in phenylalanine under drought stress implies a deficiency of the biosynthetic precursor of phenolics and flavonoids, which may lead to a decrease in phenolics and flavonoids. For tryptophan, we found that drought decreased tryptophan content. Ghorbanpour et al. [36] supported this result, and pointed out that tryptophan was reduced in barley under moderate and severe drought stress. Osmotic stress also decreased tryptophan levels in *Arabidopsis* [37]. Tryptophan is an important precursor for the biosynthesis of auxin in plants [37], and its reduction implies insufficient auxin secretion and growth restriction, consistent with our previous findings that drought reduced biomass accumulation in *B. napus* [9].

When exposed to drought, fullerol treatment had no effects on the accumulation of phenylalanine and tryptophan, but reduced the contents of glutamate, proline, and arginine in *B. napus* seedlings. This metabolic result was consistent with the RNA-seq result. For example, drought with fullerol treatment up-regulated the expression of glutamate catabolic genes *GSH3*/*GLN1-4*/*GLN1-1*, and down-regulated the expression of proline biosynthetic genes *P5CS1*/*P5CS2*, in comparison with drought alone. The reduction in proline, caused by fullerol under drought, is probably due to the fact that fullerol treatment can increase the leaf RWC, which means that plants do not need to biosynthesize proline for osmotic adjustment; thus plants can invest more in the photosynthetic response or other drought tolerance pathways. In addition, the contents of glutamate and proline in the exogenous fullerol with drought treatment were similar to those observed in the well-watered treatment, suggesting that the application of fullerol may reduce the requirement for plants to synthesize proline in response to drought.

It is worth noting that our previous work reported that fullerol treatment enhanced the leaf RWC under drought stress [9]. However, in this study, we found that exogenous fullerol did not accumulate the monosaccharides and specific amino acids, such as proline, in response to drought. The improvement in leaf RWC by fullerol may be because fullerol is able to serve as an additional intercellular water supply, rather than because of the accumulation of monosaccharides and specific amino acids to maintain water potential in leaves of *B. napus* under soil drying [8,9].

### 3.3. Secondary Metabolite Metabolism

Drought stress induces oxidative stress in plants to produce ROS, leading to membrane lipid peroxidation, protein denaturation, and DNA damage. The plants can reduce free radical damage in cells by increasing antioxidants. The phenylpropane metabolic pathway is a key metabolic pathway for secondary metabolites in plants [38]. The phenolics and flavonoids produced by the phenylpropane metabolic pathway are typical natural antioxidants in plants that resist environmental stresses [39,40]. Some studies have shown that drought increased the accumulation of phenolics and flavonoids, which helped to reduce ROS in plants during drought [41,42]. However, in the present study, we found that water deficit decreased the contents of phenolics and flavonoids such as hyperoside, peonidin 3-O-glucoside cation, quercetin 3′-methyl ether, 3-hydroxy-4-methoxycinnamic acid, and trans-3-coumaric acid. Among them, trans-3-coumaric acid is involved in the phenylpropanoid metabolic pathway. Several studies supported this result and Hernández et al. [43] showed that flavanols, including epicatechin gallate and epigallocatechin gallate, were reduced by drought stress in tea. A reduction in the concentration of rutin (flavonoid) was found in leaves of *Bupleurum chinense* DC exposed to water deficit [44]. Our results also showed that phenylalanine, one of the biosynthetic precursors of phenolics and flavonoids, was inhibited by drought, which may lead to a reduction in phenolics and flavonoids. In this study, the reduction in phenolics and flavonoids may be because these substances, in the leaves, are consumed to maintain primary metabolic functions, such as monosaccharides or prolines during drought stress [44].

Under drought stress, exogenous application of fullerol increased the contents of phenolics and flavonoids such as luteolin, trans-3-coumaric acid, chlorogenic acid, and 3-hydroxy-4-methoxycinnamic acid. Among them, chlorogenic acid and luteolin are involved in the “flavonoid biosynthesis” pathway. Additionally, fullerol elevated the expression of related biosynthetic genes under drought conditions. As an example, genes (*PAL1*/*PAL2*) encoding enzymes related to the synthesis of trans-3-coumaric acid were up-regulated by fullerol under drought. Furthermore, the levels of phenolics and flavonoids in the fullerol with drought treatment remained lower than in the well-watered treatment. These findings indicated that fullerol alleviated the inhibitory effects of drought on the accumulation of phenolics and flavonoids.

## 4. Materials and Methods

### 4.1. Plant Materials and Growth Conditions

This experiment was conducted in a controlled growth chamber with a 14 h photoperiod (07:00–21:00 h BST) and day/night temperature of 25/18 °C, in the Experimental Station of the Oil Crops Research Institute, Chinese Academy of Agricultural Sciences, Wuhan China. We selected uniform *B. napus* seeds of the Zhongshuang 11 genotype and surface-sterilized the seeds using 0.2% HgCl_2_ for 10 min and washed them with distilled water. A mixture of a loamy clay soil and vermiculite (soil:vermiculite=2:1, *v*/*v*) (1 kg) was used to fill each plastic container. We sowed eight seeds and thinned them into four seedling plants in every pot. For the initial 20 days after sowing (DAS), all pots were watered daily by weight to maintain soil water content (SWC) at 75–80% field capacity (FC). Then, two water treatments were performed: 1) plants were maintained at 80% FC daily; and 2) pots were controlled at 80% FC during the initial 20 days, and then SWC was reduced to 30% FC at 25 DAS. A small amount (5 mL) of distilled water (0 mg L^−1^ fullerol) or 100 mg L^−1^ fullerol (C_60_(OH)_27_, purity >99.9%) was applied to leaves of seedlings in each pot every other day, during 21 to 25 DAS. Fullerol synthesized from fullerene C60 using the O_2_/NaOH approach, according to the method of Li et al. [45], was purchased from the Suzhou Dade Nanotechnology Co. Ltd., Suzhou, China. The treatment combinations were as follows: sufficient water condition + 0 mg L^−1^ fullerol (Check, CK), drought + 0 mg L^−1^ fullerol (D), and drought + 100 mg L^−1^ fullerol (D + F). The 3^rd^ leaves of seedlings in the three treatments were sampled for RWC, RNA-seq, and metabolomic analysis at 25 DAS. The aboveground tissues for all treatments at 30 DAS were collected for the measurement of biomass.

### 4.2. RNA-Seq Analysis

#### 4.2.1. RNA Extraction and Quantification

Total RNA isolation from leaves was carried out using TRIzol reagent (Invitrogen, Burlington, ON, Canada). RNA degradation and contamination were determined on 1% agarose gels. The quantification and qualification of RNA were then checked using a Nano Photometer spectrophotometer (IMPLEN, Westlake Village, CA, USA), a Qubit RNA Assay Kit in a Qubit 2.0 Fluorometer (Life Technologies, Carlsbad, CA, USA), and an Agilent 2100 Bioanalyzer (Agilent Technologies, Santa Clara, CA, USA). The cDNA library construction and sequencing were conducted at a commercial service company (Novogene, Beijing, China; http://www.novogene.com, accessed on 20 September 2017). Three biological replicates were used in RNA-seq experiments.

#### 4.2.2. Data Processing

Raw reads were cleaned by removing low-quality sequences, and those containing adapter or poly-N sequences. The index of the reference genome was built using Bowtie 2.2.3. TopHat 2.0.12, which is a fast-mapping tool based on generating a database of splice junctions. This was used to align clean paired-end reads to the reference genome. The clean reads were mapped to the *Brassica napus* genome (http://brassicadb.org/brad/datasets/pub/Genomes/Brassica_napus/, accessed on 8 October 2017) [46]. HTSeq 0.6.1 was used to summarize read counts mapped to each gene. The gene expression levels were quantified by fragments per kilobase of transcript per millions reads (FPKM) that eliminate the influence of gene lengths, and sequencing discrepancies.

DESeq R package (1.18.0) was used to identify the DEGs between two groups with three replications. For controlling the false discovery rate, the resulting *p* values were adjusted using Benjamini and Hochberg’s procedure. Genes with an adjusted *p* value less than 0.05, identified by DESeq, were regarded as being differentially expressed [47,48]. The DEGs were then used to conduct functional annotation, including GO and KEGG analysis. GO seq R package was used to implement the functional enrichment analysis of GO. KOBAS software (KOBAS, Surrey, UK) was used to conduct KEGG pathway enrichment analysis, which can calculate the total number of DEGs involved in specific pathways.

#### 4.2.3. qRT-PCR Analysis

To validate RNA-seq results, we selected 22 genes to explore their expression patterns using qRT-PCR analysis. Three independent biological replicates were conducted, and each biological group was repeated three times. Total RNA samples were isolated from leaves using TRIzol reagent (Invitrogen, Burlington, ON, Canada). One μg of total RNA was reverse-transcribed by RevertAid^TM^ First Strand cDNA Synthesis kit (Fermentas, Burlington, ON, Canada). We used the Bio-Rad Real-Time System (BioRad, Hercules, CA, USA) to conduct qRT-PCR in 50 μL of reaction mixture, including: 25 μL 2× SYBR^®^ Premix Ex Taq^TM^ II (Takara, Kusatsu, Japan), 2 μL of each PCR forward and reverse primer for the selected gene, 1 μL of 50 × ROX reference dye, 4 μL of cDNA template, and 16 μL of dd H_2_O. The amplification conditions of PCR were as follows: one cycle of 95 °C for 30 s, 40 cycles of 95 °C for 5 s, and 60 °C for 30 s. The transcript level of the β-actin gene was set as a control. We listed the primer sequences for the selected genes in Table S2.

### 4.3. Metabolomic Analysis

#### 4.3.1. Metabolite Extraction and LC-MS Conditions

Untargeted metabolomic profiling was conducted by Novogene company (Beijing, China). The leaf tissues (50 mg) were extracted in 1 mL of methanol:methylcyanide:water mixture (2: 2: 1, *v*/*v*/*v*) for 1 h at –20 °C, centrifuged at 13,000 rpm for 15 min at 4 °C to obtain the supernatant, freeze-dried, and stored at –80 °C. A total of 100 μL of methylcyanide:water mixture (1:1, *v*/*v*) was added to the dried sample, and the solution was then vortexed for 30 s and centrifuged at 14,000 rpm for 15 min at 4 °C. The supernatant was transferred into an LC vial for nontargeted global ultra-performance liquid chromatography-quadrupole time-of-flight mass spectrometry (UPLC-Q-TOF-MS) analysis. For quality control, an equal mixture of all samples was taken. There were six biological replicates for each treatment using UPLC-Q-TOF-MS analysis.

We used Agilent 1290 Infinity LC (Agilent Technology, Santa Clara, CA, USA) equipped with an Acquity UPLC HSS T3 column of 2.1 mm × 100 mm (Waters, Milford, MA, USA) to separate the production. The temperature of the column was set at 25 °C and the flow rate was 0.3 mL min^−1^. Mobile phase A was water/25 mM ammonium acetate/25 mM ammonia, and mobile phase B was acetonitrile. A gradient elution program consisted of 95% B at 0–0.5 min, 95–65% B at 0.5–7 min, 65–40% B at 7–8 min, 40% B at 8–9 min, 40–95% B at 9–9.1 min, and 95% B at 9.1–12 min. The separated components were detected in the positive and negative electrospray ionization modes using a Triple-TOP 5600 mass spectrometer (AB SCIEX, Concord, ON, Canada). Ion source gas 1 and 2 and curtain gas were set at 60, 60, and 30, respectively. The TOF MS scan m/z range was 60–1200 Da, and the MS/MS scan m/z range was 25–1200 Da. The accumulation time was 0.15 s/spectra for TOP MS, and 0.03 s/spectra for MS/MS. The source temperature was 600 °C and the IonSpray voltage was set at ± 5500 V.

#### 4.3.2. Multivariate Data Processing

Multivariate methods including PCA, and partial least-squares discriminant analysis (PLS-DA) were used for normalized data analysis [49,50,51]. The inclusion/exclusion criteria of: (1) variable importance in projection (VIP) > 1.0; and (2) *p*-value < 0.05 were performed for the identification of the metabolites [52]. Metabolites that reached these criteria were marked as significantly differential metabolites. The significantly differential metabolites obtained from each comparison group underwent KEGG ID mapping, and were submitted to the KEGG website for relevant pathway analysis. 

### 4.4. Statistical Analyses of Other Data

Statistical analyses for qRT-PCR data were carried out using one-way ANOVA, and Duncan’s multiple range test was performed to compare the significant differences among treatments at *p* = 0.05 level.

## 5. Conclusions

In this study, we investigated the molecular and metabolic mechanisms induced by fullerol in enhancing drought tolerance in *B. napus* seedlings using the transcriptomic and metabolomic analyses. The results show a correspondence between profile changes in genes, and profile changes metabolites. The DEGs and differentially accumulated metabolites triggered by drought or fullerol were commonly enriched in KEGG pathways associated with carbohydrate metabolism, such as “carbon metabolism”, amino acid metabolism such as “biosynthesis of amino acids”, and secondary metabolite metabolism such as “biosynthesis of secondary metabolites”. We analyzed the DEGs and differential metabolites in these KEGG pathways and found that *B. napus* seedlings subjected to soil drying exhibited a high accumulation of primary metabolites, and inhibited the accumulation of secondary metabolites. The accumulated primary metabolites, including monosaccharides (e.g., mannose and myo-inositol) and specific amino acids (e.g., proline), can promote the osmotic adjustment ability in leaves of *B. napus* seedlings in response to drought. The results further showed that fullerol treatment could reverse the inhibitory effects of drought on the accumulation of secondary metabolites, such as phenolics and flavonoids (e.g., luteolin and trans-3-coumaric acid), but had no impacts on the accumulation of osmotic adjustment substances (e.g., monosaccharides and specific amino acids) to enhance drought tolerance in *B. napus*.

## Data Availability

Raw data have been deposited in the Gene Expression Omnibus (GEO) international repository, and the GEO accession number is GSE216368.

## Supplementary Materials

The following supporting information can be downloaded at: https://www.mdpi.com/article/10.3390/ijms232315304/s1, Figure S1: KEGG pathway analysis of differentially expressed genes in leaves of *B. napus* after drought and fullerol treatment. Top 20 enriched KEGG pathways for the up-regulated (a, c, e) and down-regulated (b, d, f) genes between drought (D) and check (CK, sufficient water condition) (D vs. CK), between drought with fullerol (D + F) and check (CK) (D + F vs. CK), and between fullerol and control in the drought treatment (D + F vs. D) are given separately; Figure S2: Principal component analysis (PCA) clustering based on metabolome data in leaves of *B. napus* under water and fullerol treatment. CK: check (sufficient water condition); D: drought; F: fullerol. (a) Group D vs. CK. (b) Group D + F vs. CK. (c) Group D + F vs. D; Table S1: Primers for quantitative real-time PCR; Table S2: The data obtained from sequencing different samples in leaves of *B. napus* under water and fullerol treatments. CK: check (sufficient water condition); D: drought; F: fullerol; Supplementary Materials File S1: Differential metabolites identified under drought (D) in comparison with check (CK: well-watered condition) in leaves of *B. napus*; Supplementary Materials File S2: Differential metabolites identified under fullerol treatment in drought-treated plants (D + F) in comparison with check (CK, well-watered condition) in leaves of *B. napus*; Supplementary Materials File S3: Differential metabolites identified under fullerol treatment in drought-treated plants (D + F) in comparison with drought alone (D) in leaves of *B. napus*; Supplementary Materials File S4: KEGG pathways of metabolites in leaves of *B. napus* in a comparison between drought (D) and check (CK, sufficient water condition); Supplementary Materials File S5: KEGG pathways of metabolites in leaves of *B. napus* in a comparison between drought with fullerol treatment (D + F) and check (CK, sufficient water condition); Supplementary Materials File S6: KEGG pathways of metabolites in leaves of *B. napus* in a comparison between drought with fullerol treatment (D + F) and drought alone (D); Supplementary Materials File S7: The common KEGG pathways based on metabolite-transcript integration using transcriptome and metabolome datasets in leaves of *B. napus* in a comparison between drought (D) and check (CK, sufficient water condition); Supplementary Materials File S8: The common KEGG pathways based on metabolite-transcript integration using transcriptome and metabolome datasets in leaves of *B. napus* in a comparison between drought with fullerol treatment (D + F) and check (CK, sufficient water condition); Supplementary Materials File S9: The common KEGG pathways based on metabolite-transcript integration using transcriptome and metabolome datasets in leaves of *B. napus* in a comparison between drought with fullerol treatment (D + F) and drought alone (D); Supplementary Materials File S10: The differentially expressed genes mentioned in Figure 6 with assay names of gene ID in leaves of *B. napus* in a comparison between check (sufficient water condition), drought, and drought addition with fullerol.
