# Supplementary material for: Transcriptomic and Metabolomic Analyses Reveal That Fullerol Improves Drought Tolerance in Brassica napus L
Source: Int J Mol Sci. 2022 Dec 4;23(23):15304. doi: 10.3390/ijms232315304 (PMC9740425; doi:10.3390/ijms232315304)
Supplement: Supplementary file 1 [file ijms-23-15304-s001.zip › Supplementary file legends.pdf]

Figure S1. KEGG pathway analysis of differentially expressed genes in leaves of *B. napus* after drought and fullerol treatment. Top 20 enriched KEGG pathways for the up-regulated (a, c, e) and down-regulated (b, d, f) genes between drought (D) and check (CK, sufficient water condition) (D vs. CK), between drought with fullerol (D + F) and check (CK) (D + F vs. CK), and between fullerol and control in the drought treatment (D + F vs. D) are given separately.

Figure S2. Principal component analysis (PCA) clustering based on metabolome data in leaves of *B. napus* under water and fullerol treatment. CK: check (sufficient water condition); D: drought; F: fullerol. (a) Group D vs. CK. (b) Group D + F vs. CK. (c) Group D + F vs. D.

Table S1. Primers for quantitative real-time PCR.

Table S2. The data obtained from sequencing different samples in leaves of *B. napus* under water and fullerol treatments. CK: check (sufficient water condition); D: drought; F: fullerol.

Supplementary Materials File S1. Differential metabolites identified under drought (D) in comparison with check (CK: well-watered condition) in leaves of *B. napus*.

Supplementary Materials File S2. Differential metabolites identified under fullerol treatment in drought-treated plants (D + F) in comparison with check (CK, well-watered condition) in leaves of *B. napus*.

Supplementary Materials File S3. Differential metabolites identified under fullerol treatment in drought-treated plants (D + F) in comparison with drought alone (D) in leaves of *B. napus*.

Supplementary Materials File S4. KEGG pathways of metabolites in leaves of *B. napus* in a comparison between drought (D) and check (CK, sufficient water condition).

Supplementary Materials File S5. KEGG pathways of metabolites in leaves of *B. napus* in a comparison between drought with fullerol treatment (D + F) and check (CK, sufficient water condition).

Supplementary Materials File S6. KEGG pathway analysis of metabolites in leaves of *B. napus* in a comparison between drought with fullerol treatment (D + F) and drought alone (D).

Supplementary Materials File S7. The common KEGG pathways based on metabolite-transcript integration using transcriptome and metabolome datasets in leaves of *B. napus* in a comparison between drought (D) and check (CK, sufficient water condition).

Supplementary Materials File S8. The common KEGG pathways based on metabolite-

transcript integration using transcriptome and metabolome datasets in leaves of *B. napus* in a comparison between drought with fullerol treatment (D + F) and check (CK, sufficient water condition).

Supplementary Materials File S9. The common KEGG pathways based on metabolite-transcript integration using transcriptome and metabolome datasets in leaves of *B. napus* in a comparison between drought with fullerol treatment (D + F) and drought alone (D).

Supplementary Materials File S10. The differentially expressed genes mentioned in figure 6 with assay names of gene ID in leaves of *B. napus* in a comparison between check (sufficient water condition), drought, and drought addition with fullerol.
