# Supplementary material for: Transcriptomic and Metabolomic Analyses Reveal That Fullerol Improves Drought Tolerance in Brassica napus L
Source: Int J Mol Sci. 2022 Dec 4;23(23):15304. doi: 10.3390/ijms232315304 (PMC9740425; doi:10.3390/ijms232315304)
Supplement: Supplementary file 1 [file ijms-23-15304-s001.zip › Tables S1.pdf]

Table S1. Primers for quantitative real-time PCR.

Gene	Sequence (5′-3′)	
	Forward primer	Reverse primer
GAE6	CCGAACGCAAGACAATCAAGAAG	CCAACGCACCGACACATCC
GAPC	CGTCCACTCTATCACTGCTACTC	GCGGCTCTTCCACCTCTCC
PME3	AATCCTTCTCAAACAACCGTAAACTC	CCGTTAGCAGCCACAGTAGC
NADP-ME1	TATCCATCCTTCTCTTCCATCCG	GCTACTCTCCGCACACTTGAC
PGL1	GAGGTGAAGGACGATTGGGTTAC	TAGCAGGCAGGGAAAGAGAGC
BXL5	GTGTAGAGGAAGGAGATGTGAGTAG	ATAACGCCACGAAGGAGATTAGG
SPS2	TGGTGACTCCGCTGCTCTTC	ACACAACTCCTCTGCCTCTATCC
GLN1-4	TCTGGGTCGCTCGTTACATCC	CGTCGCTCGTTGCCTTCAC
GLN1-1	TTTAGCCACCCTGATGTTGTAGC	CTAATGTTGATGCCAGCGTATAAGC
P5CS2	GGTGTCTTCTGGTGCGGTTG	CCTGAAATCCTTATCTCTGAAACTGC
P5CS1	TTCCACAACGCAAGCACAAG	CCACCTCAGCACCAAGTCC
GPX1	GCTTTCCCTTGTAATCAGTTCGG	TGCTCGGTCCATTCACATCG
GSTF3	CTCCCAGCAGACTCCAAGAAC	GCCTAGCCTCGTAGACATCAAG
APX1	AGCGAAGATGACGAAGAACTACC	AACCTAAGAGCAATGTGGAGACC
