# Supplementary material for: Transcriptomic and Metabolomic Analyses Reveal That Fullerol Improves Drought Tolerance in Brassica napus L
Source: Int J Mol Sci. 2022 Dec 4;23(23):15304. doi: 10.3390/ijms232315304 (PMC9740425; doi:10.3390/ijms232315304)
Supplement: Supplementary file 1 [file ijms-23-15304-s001.zip › Tables S2.pdf]

Table S2. The data obtained from sequencing different samples in leaves of *B. napus* under water and fullerol treatments. CK: check with sufficient water condition; D: drought; F: fullerol.

Sample name	Raw reads	Clean reads	Q30 (%)	Total mapped	Uniquely mapped	Number of FPKM (>1)
CK1	58154238	56213860	91.3	88.1%	84.5%	47759
CK2	58247532	56425320	93.0	88.9%	85.2%	47806
CK3	54015134	52343136	93.1	88.8%	85.2%	48033
D1	58896344	57028610	93.0	88.8%	84.5%	46123
D2	56954304	55101912	92.3	88.8%	84.6%	46202
D3	58074628	56427842	93.1	88.5%	84.4%	46893
D + F1	56241264	54465614	93.0	89.2%	85.1%	47292
D + F2	64665902	62866522	93.2	88.9%	84.7%	47984
D + F3	57748296	55892760	93.1	88.4%	84.5%	48924
