# Supplementary figures and images for: Transcriptomic and Metabolomic Analyses Reveal That Fullerol Improves Drought Tolerance in Brassica napus L
Source: Int J Mol Sci. 2022 Dec 4;23(23):15304. doi: 10.3390/ijms232315304 (PMC9740425; doi:10.3390/ijms232315304)

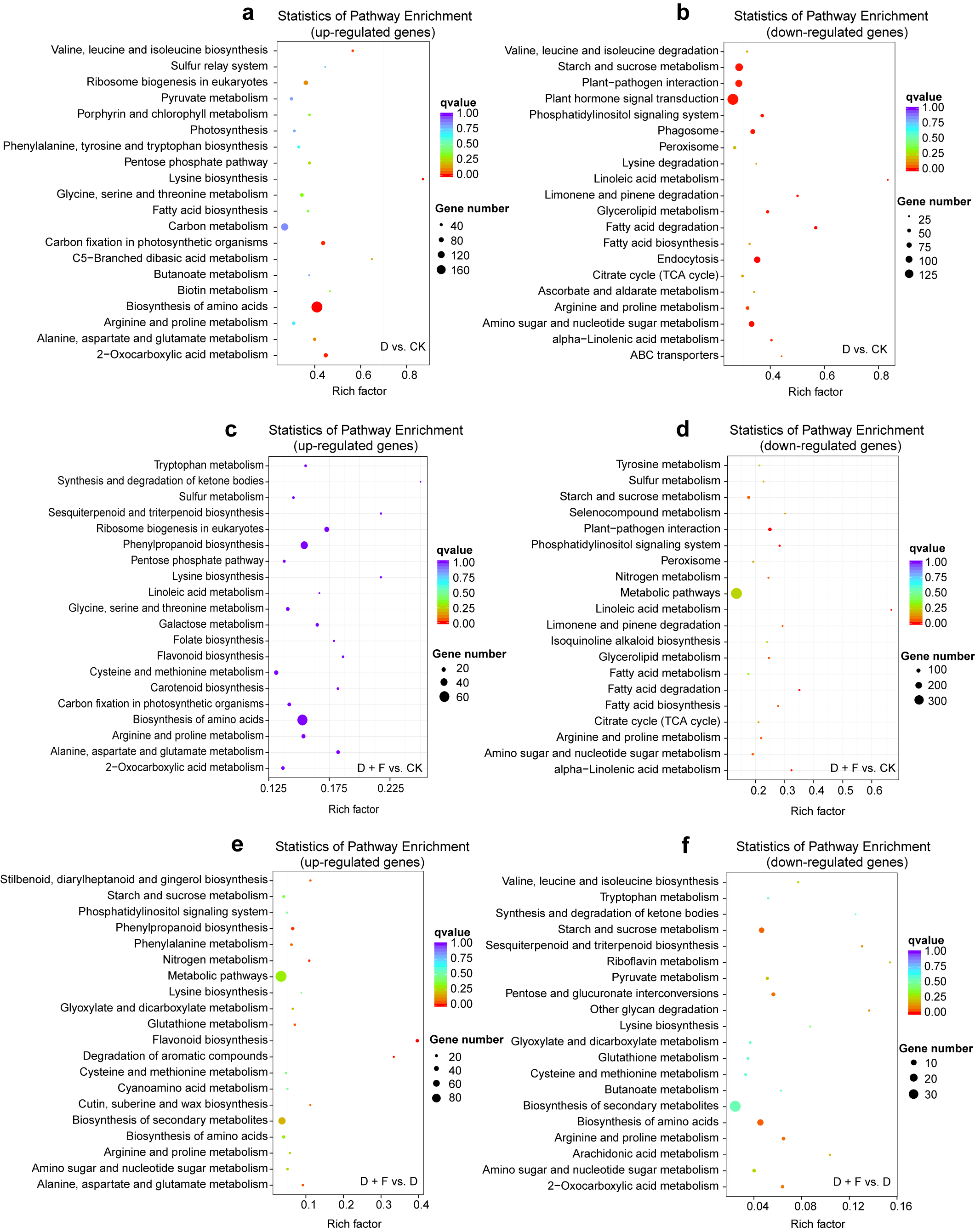

Supplement: Supplementary file 1 [file ijms-23-15304-s001.zip › Figure S1.tif]

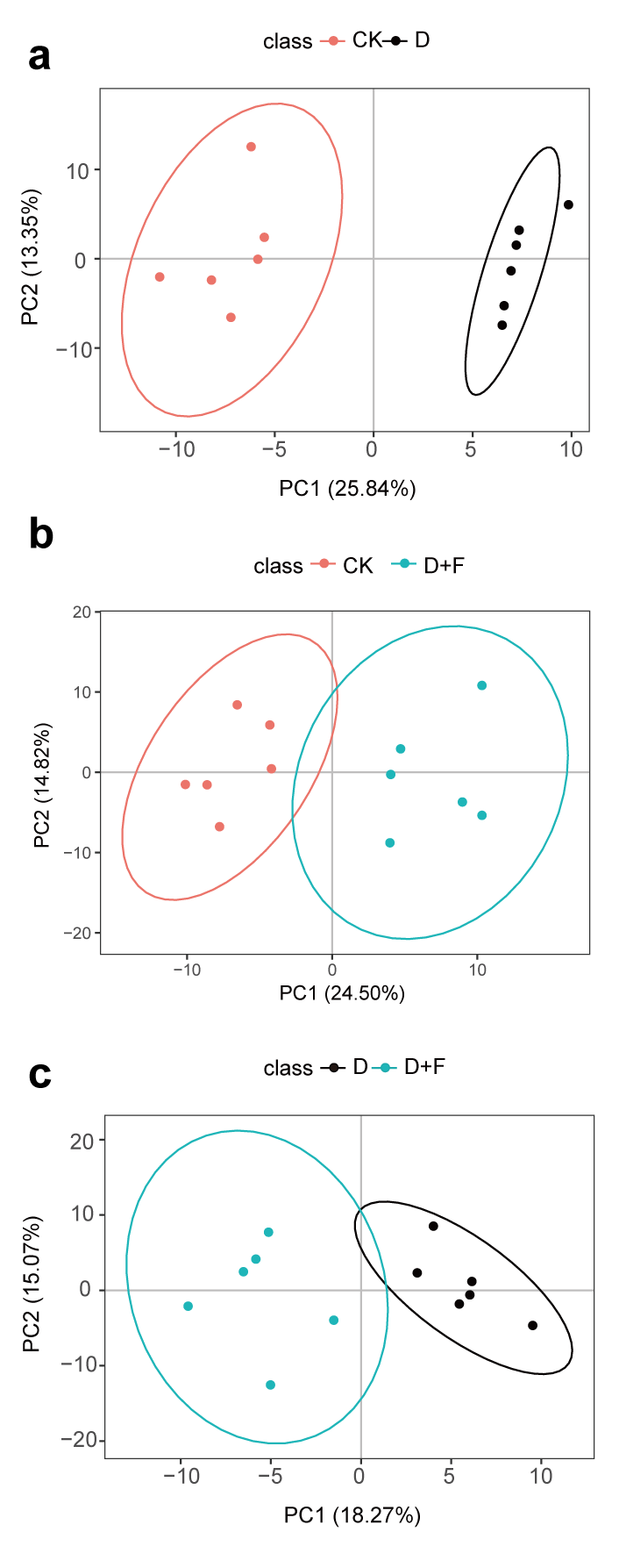

Supplement: Supplementary file 1 [file ijms-23-15304-s001.zip › Figure S2.tif]
